# Supplementary material for: Changes in access to viral load testing, incidence rates of viral load suppression and rebound following the introduction of the ‘universal test and treat’ guidelines in Cameroon: A retrospective follow-up analysis
Source: PLOS Glob Public Health. 2024 Apr 16;4(4):e0003042. doi: 10.1371/journal.pgph.0003042 (PMC11020606; doi:10.1371/journal.pgph.0003042)
Supplement: Attachment — Submitted filename: Response to Reviewers.docx [file pgph.0003042.s002.docx]

**Manuscript Number: PGPH-D-23-02088**

**Changes in access to viral load testing, incidence rates of viral load suppression and rebound following the introduction of the ‘Universal Test and Treat’ guidelines in Cameroon: a retrospective follow-up analysis.**

**AUTHORS RESPONSE TO REVIEWERS’ COMMENTS**

We thank the Editor and Reviewers for their time and effort to review our manuscript and provide constructive comments to improve the scientific quality of our manuscript. We hereby provide our responses to the comments raised to the best of our ability.

**EDITOR'S COMMENT: In addition to the reviewers' comments, I have a comment for your response: I noticed that you recently published a similar paper entitled: "The effect of the Universal Test and Treat policy uptake on CD4 count testing and incidence of opportunistic infections among people living with HIV infection in Cameroon: a retrospective analysis of routine data". Please provide a sound scientific rationale for the submitted work and clearly reference and discuss the existing literature.**

*In our series on UTT and its effect on the components of the cascade of HIV care (from diagnosis through linkage and treatment to viral load suppression) we looked at CD4 testing and opportunistic infections as rightly mentioned. In this manuscript, we focused on the ultimate and most desirable outcome in HIV management. We conducted a literature review on the clinical trials and observational studies on the efficacy and effectiveness of UTT on viral load as indicated in reference 4 to 23.*

**Reviewer #1: Review 1**

**Title: Changes in access to viral load testing, incidence rates of viral load suppression and rebound following the introduction of the ‘Universal Test and Treat’ guidelines in Cameroon: a retrospective follow-up analysis.**

**Abstract**

**Background: I guess retention also is a desirable outcome in HIV care. I am wondering why the authors did not look at retention as while. Why didn’t the authors also look at the clinical and demographic factors associated with VLS before and after UTT, informing cardinal for targeted intervention? If such information is available, it will be nice to see such an analysis.**

*We thank the reviewer for his interest in retention given its pivotal role in HIV care. In fact, we looked at the effect of UTT across the cascade of HIV care including retention but in detail in a separate article published in PAMJ. Given word count limitations we thought of VLS in a separate manuscript. We do not have data on clinical and demographic factors observed separately for each era.*

**Methods: authors can consider segregating the pre- and post-UTT period. E.g before (between 2002 and 2015) and after (between 2016 and 2020), you can also include the months. Was the data collected from both children and adults? Include the information on points through which data were collected e.g. 6, 12, 24 months etc and state the tool used to collect the data. The period before UTT was longer than the after. Why didn’t the authors have an equal follow-up period? What was the primary outcome? If viral suppression, how was it defined? I encourage the authors to include such information. In the last sentence, authors can specify the VL events (VLS and VLR). VLR should be stated in the aim.**

*These suggestions have been included in the abstract as advised. We are aware that the periods of follow-up between and within the periods are unequal (staggered entry) reason why we measured the incidence rate as opposed to cumulative incidence to account for person-time spent during follow-ups.*

**Results: first paragraph, was there a statistical difference in the proportion of VLT? Does it mean data were only collected at two points before and after UTT i.e. 6 and 12 months? If yes, how was it done given that data was collected between 2002 and 2020? What were the inclusion criteria for participants in the pre-and post-UTT? I thought that each individual was followed for a specific period before and after UTT. OR did you follow up with everyone and UTT was part of the variables? If yes, what were the inclusion criteria as of 2002? What was the average overall follow-up period? And also segregate it for before and after UTT. CI should be written as “confidence interval (CI)” since it appears for the first time. Do the same for “aHR”. Say “The incidence of VLR was more than twice as high in the UTT group than in the pre-UTT group (aHR = 2.32, 95%CI: 1.30 – 4.13) after adjusting for …………………..”. I encourage authors to indicate the proportions of VLS. I did not see the results on VLR.**

*There was a statistical difference in the proportions as indicated by the p-values we have now included in the sentence. Patients enrolled before 2016 were included in the pre-UTT group while those enrolled thereafter were included in the post-UTT group. Everyone was then followed until censoring. We have added the meanings of CI and aHR. Rates of VLS and VLR have been included as well though this hasmakes the abstract a bit longer than the words count allowable.*

**Conclusion: Where are the concerns coming from over the durability of VLS? What informs the concerns? I encourage the authors to highlight a recommendation/s based on their findings.**

*VLR was higher in the UTT group thus raising concerns about durability in the era of UTT.*

Introduction

**- “Achieving the third 95 (viral load suppression - VLS) is the ultimate and most desirable target in the care of PLHIV and this can only be attained by an effective ART”, cite. And please note that retention is also cardinal in achieving viral suppression.**

*Cited included.*

**- 3 years should be written as “three years or three (3) years”.**

*Revised accordingly.*

**- “Viral suppression defined as a viral load<1000 copies/ml according to the WHO, can only be recognised or achieved when clients can assess viral load testing (VLT)”, I don’t think viral load testing can help to achieve VL <1000, I do agree on the recognition part. Probably retention is one of the factors imperative to achieving VLS. I propose you delete “or achieved” and information on characteristics important in achieving VLS.**

*The word ‘achieved’ has been deleted.*

**- “VLT gives clients a measure of understanding, control, and motivation to adhere to treatment and understand their HIV infection”, back this information with scientific evidence. Note that other clients do not bother about VLT as long as they can get their meds. Based on my experience, vein puncture does not sit well with most ART clients.**

*Citation has been added.*

**- The gap is not really coming out; does it mean viral load coverage and VLS were unknown before and after UTT in Cameroon? See our publication where we also looked at VLS https://pubmed.ncbi.nlm.nih.gov/37900026/.**

**- The authors should do more literature review on VLT, VLS and VLR comparing the before and after UTT.**

*We do not know what VL monitoring looks like after UTT thus the need for this study. We have extensively (but probably not exhaustively as new evidence emerge) conducted a literature review as indicated in citations* ***4 to 17*** *on VLS and UTT. We thank the Reviewer for their article on a similar study and we have now cited their work as well as far as VL is concerned.*

**Methods**

**- I encourage the authors to have a “Study setting” separate from viral load monitoring. I think viral load monitoring can be under “Data collection and study procedure” OR it can have its section. Indicate the study design before the viral load monitoring BUT I don’t understand why viral load monitoring has such details. Did the authors use retrospective data? If yes, the sample size estimation, data abstraction process (sampling method/s), data quality control, definitions of outcomes, data analysis, and ethical consideration would be of great concern to me. If the authors conducted a prospective study in the period after the test and treat policy, the “Study procedure” section would address the issues of viral load monitoring. However, the abstract has already stated that it was a retrospective analysis. General guidance on the flow of methods section: “1) study setting 2) study design and population 3) sample size estimation, 4) data abstraction process (sampling method/s), 5) data quality control, 6) definitions of outcomes, 7) data analysis, and 8) ethical consideration”. The whole issue of viral load monitoring is confusing. The definitions for VLS, VLR and viral load coverage/testing can be defined under the section “Definitions of outcomes or operational definitions”.**

*We do agree with the reviewer on the flow of the methods section.* *Given that testing, linkage, treatment, and viral load monitoring guidelines are adapted to specific settings, we thought it would be intuitive to describe the setting alongside their procedures on how VLT is being conducted, at what intervals, the definitions of VLS and VLR used, etc. We have though presented all the information requested though subtle changes in the order of presentation are observed but still in the lens of PLOS guidelines.*

**- Figure 1, I don’t seem to fit it anywhere in the document. Maybe authors can consider having a flowchart for inclusion criteria so it can show numbers analysed for both before and after UTT.**

*The flow chart incorporates elements of a cohort design from inclusion based on exposure to UTT, through follow-ups to viral load testing outcomes.*

**- “while those enrolled after the year 2006 were included in the UTT (exposed) group.”, I guess authors meant 2016 NOT 2006. Please correct accordingly. The periods before (2002 to <2016) and after (2016 to 2020) UTT are not similar. Is there a special reason for this?**

*2016 rightly corrected. The pre- and post-UTT periods are not the same indeed as per the time the study was conducted. However, by conducting time to event analyses, we accounted for time spent in each period.*

**- Authors should state whether they included clients who were just starting ART at baseline or not.**

*We conducted the study in 2022 but included only patients who were enrolled up to 2020 to allow for the time to conduct the routine VLT at 6, 12 months and thereafter.*

**- Authors should state the follow-up periods e.g. at baseline, 6, 12, 24 months**

*We have stated the follow-up periods in the flow chart and in the description of viral load monitoring guidelines.*

**- “The groups were then followed up retrospectively until their first and repeat viral load measurements were done to compare their median time to VLT, and their rates of VLS and VLR.” Since authors were looking for this why didn’t they look at individuals initiating ART before and after UTT and follow them up for a period of one year for each period? I can’t understand why authors had to go back to 2002. If it is the issue of VLR they would have extended the period to 2 or 3 pre- and post-UTT.**

*We did just that but given the fact that all patients did not start ART at the same time and did not all get tested for VL at the required times, it was necessary to compare median times to VLT as was as calculate incidence rates instead of incidence proportions because the latter assumes that all patients had an equal follow-up period of observation.*

**- Data collection and study variables: authors should state what type of data were collected at what period e.g. baseline, 6 and 12 months.**

*All sociodemographic and clinical data were collected at baseline while viral loads were collected at various times during follow-up of individual patients though we did censoring at 6 and 12 months to determine coverage of VL and compared median times to serial VLT events.*

**- Authors should be detailed on how data were collected so we can connect it with the data analysis plan.**

*In this retrospective data collection, attempting to describe how each variable* *was measured will be speculative because we merely used a data extraction form* *to collect routine service data as transcribed in individual patient medical record.*

**- “The data set was explored for logical inconsistencies, illegal codes, omissions and improbabilities by tabulating, summarising, describing, and plotting variables”, was this part of data cleaning processing? This is what is usually done in the background and rarely mentioned in the manuscript. You may need to remove this part.**

*Deleted as advised.*

**- For missing values, authors may consider an imputation method.**

*Not considered*

**- “Viral load coverage was defined by the proportion of patients accessing a routine VL testing performed at the time it was due within the study period”, should be part of the “Operational definition”.**

*Moved to the VL monitoring section.*

**- Say “Schoenfeld residuals was used for checking and testing the proportional-hazards assumption”.**

*Rephrased as suggested.*

**Results**

**- Table 1, first row should include the total number (%) for individuals before and after UTT.**

*Due missingness, this number slightly differs across variables and is thus indicated as the total for each variable***.**

**- “545 (33.54%) initiated on ART on the same day of HIV diagnosis”, this information is not there in Table 1.**

*The 545 PLHIV on SDI have been mentioned in the table but have been further* *categorised between the pre- and post-UTT periods.*

**- “Participants enrolled under the UTT era were more likely to start ART on the same day of their HIV diagnosis (67.24%)”, this is not a surprising finding to due guideline issue as compared to the period before UTT. Hope the discussion have taken care of this.**

*This was to indicate whether uptake of SDI policy was effectively rolled out.*

**- Table 1, please countercheck because some of the variable they don’t add up to 100% e.g. marital status. Check all the variable and revise accordingly.**

*We have checked and revised all the variables affected by rounding up.*

**- Table 1, why was age categorized in that way? You may want to categorize age based on some similarity with regards to sociodemographic or other factors responsible for the outcome. I don’t think there are similarities in VLS between adolescent and/or young adults (15 to 19 or 19 to 25 years) and those aged 25 to 35 or those aged 36 to 40 years. Check some recommendations from WHO.**

*This categorisation was based on the age distribution of the study population.*

**- Table 1, variable area of residence, it would be more understandable by other HIV program managers from other countries if it was categorized as “rural or urban”. In its current only Cameroon can benefit from the information.**

*This categorisation was a proxy indication for the distance to the clinic.*

**- Did you have any missing values in some variables, if yes, it would be nice to indicate them.**

*There were some missing values, and this was the reason why we indicated the totals of each variable (instead of the grand total on the first row)*

**- Table 1, CD4 should be categorized based on some source or clinical knowledge. Immunocompetence if am not mistaken >=500. If you want to leave the threshold of 350 define as such in operational definition section.**

*Before UTT, CD4 was used as the marker to determine when to start ART and one of such critical thresholds before 2015 was CD4<350.*

**- Same of the initiators should be written in full to avoid confusing the readers e.g. SDI, RI and DI. Sometimes too many abbreviations are confusing.**

*Revised as suggested.*

**- The narration for Table 2 should have own subheading. Do the same for Table 3.**

*The subheadings for these sub-sections of the result section have been included as Predictors of viral load suppression and Predictors of viral load rebound respectively.*

**- “Overall, the proportion of the cohort who achieved VLS was 78.60% (95%CI:75.56 –81.42) but the UTT group had a higher proportion of VLS than the pre-UTT group (85.83% vs. 71.98%, p<0.001).” which table shows this information? I suggest Table 2 and 3 should have results for a chi-square test.**

*This is indicated as a rate of VLS in Table with the 95% confidence interval and p-value.*

**- What was the primary outcome between VLS and VLR?**

*Apparently, we did not distinguish between the two as such because we were interest in changes across VL monitoring from testing, suppression to rebound.*

**- First define aHR and thereafter use the initials**

*Abbreviation has been defined accordingly.*

**- Did you collect data on the type of ART?**

*Yes, we did but so there many types of regimens for the first and second lines.*

**- What do you mean by “time and type of treatment initiation”?**

*We meant period (pre- /post UTT) and same-day or delayed initiation. ‘Time’ has been replaced with ‘period’.*

**- Table 2 and 3, the variables used for controlling the model should not be in the table. You can have a supplementary table for them.**

*Only variables which were significantly associated with the outcome in the simple regression analyses were included in the final table.*

**- What does “t” mean “of t SDI”?**

‘t’ has been removed from the sentence.

**- “Other factors significantly associated with higher VLS rates were age above 40 years, early clinical presentation, and current employment status”, narrate these results as opposed just stating like this.**

*We have now added the estimates and their 95%CI to these factors to show the strength and validity of the associations with VLS.*

**- Table 2 has no lifestyle variables.**

*Agree, these were not significant and were not included; lifestyle has been dropped from the footnote.*

**- Similarly, under this section “Universal ‘test and treat’ and changes in viral load rebound rates” some results ae not shown either in a table format or figure.**

*We are unable to detect these results.*

**Was the rate of VLR statistically different for pre- and post-UTT?**

*There was a statistically significant difference in the VLR rate between the periods: the incidence of VLR was twice as high in the UTT group than in the pre-UTT group ([adjusted Hazard ratio (aHR) = 2.32, 95%CI: 1.30 – 4.13, p = 0.004).*

**- In Table 3, all the significant variables should be narrated.**

*We have now added the estimates and their 95%CI to these factors to show the strength and validity of the associations with VLR.*

**- I didn’t see alcohol consumption in the models. Maybe instead of having a supplementary table, show all the variables in your models.**

*Only variables that were associated with the outcomes at least in the univariable regression model have been presented. Alcohol intake was not associated with any of the outcomes. It may be boring to some readers to go through a long table with insignificant results.*

**- You can also consider looking at the factors associated with VLS and VLR for Pre- and post-UTT. It will be great to see the drivers of these outcomes in the period before and after the test and treat; tables can be supplementary.**

*Nice input though but we did not collect those variables separately. We shall consider this suggestion as one of our potential research topics to work on.*

**- Include the year in your analysis if you have such information, to see the trends of VLS and VLR across the years.**

*Interesting tip, but we have been wondering which years to consider year of enrolment or of viral load testing or year of viral load results given that there are significant discrepancies in these dates for individual patients. Trends in Viral load coverage are however more feasible to construct.*

**Discussion:**

**- No need to restate the aim of the study.**

*To understand the main findings presented in the first paragraph, it’s worthwhile to mention the main objective.*

**- How was timely viral load testing calculated when individuals have a specific period to come to the clinic based on the guideline? It would be nice to see a proportion of individuals who had a viral load test at specified time intervals in line with your national guidelines.**

*We stated the viral load coverage after 6 and 12 months on ART.*

**- The first paragraph should summarize your findings in line with your aim.**

*Done.*

**- “The persistently low VLT coverage is linked to the increasing number of patients who are also accessing ART because of UTT and who eventually require a VLT”, I don’t think this is a reason enough for low VL coverage. If you have the necessary and enough human resources, equipment, testing facilities and reagents, you can still have a large proportion of those who have a VL test.**

*These resources have increased as well but do not match the demand for VLT. So, though the number of people accessing VLT has increased in absolute terms, in relative terms, coverage remains low.*

**- What do you mean by early presentation? Or which results represent early presentation?**

*Early presentation = presentation at clinical stage I or II.*

**- You can also discuss the significant findings on the factors associated with VLS and compare with previous literature like ours and many more.**

*Identifying predictors for VLS has not been our focus but these factors were examined as potential confounders for the effect of UTT on VLS. We discussed a few of them like early/late clinical presentation that we believe could explain the changes observed because they had also evolved over time.*

**- “While this study has indicated that there have been improvements in VLS under UTT, there is uncertainty as to whether UTT may potentially delay the time to viral rebound after initial viral suppression”, uncertainty by who?**

*Our data have indicated that, UTT was associated with a higher rate of VLR so raising ‘our’ concerns about VLS durability during this period. We have rephrased the sentence accordingly.*

**- How many months is “multi-month drug dispensations”?**

*Usually 3 – 6 months depending on ARV in stock.*

**- Does it mean, the authors had limited literature to compare with theirs? Authors have more probable explanations compared to the cited previous literature. I encourage the others to do more literature reviews.**

*As earlier indicated, we have reviewed a dozen of publications on VLS during UTT and have observed lots of conflicting results across settings. This inconsistency has been attributed to methodological issues and moreover by context specific enablers and barriers. Reason why we focused our discussion on context and less on comparison.*

**- Discuss the other variables significantly associated with VLR.**

*Discussed briefly in the lens of interventions to target them in this context.*

**- In the section for study limitations and strengths, take the recommendation/s to the conclusion.**

*The recommendation is specific to a limitation identified so taking down to the conclusion will not tie with the study conclusions. We have however removed the word ‘recommendation’ and replace it with ‘solution’.*

**- Interrupted time series would also be a good quasi-experimental study design to generate good estimates for your counterfactuals.**

*Suggested incorporated.*

**- Why do you expect typing errors?**

*Is a possibility when data clerks key in data.*

**- Given that this was longitudinal data, did you have LTFU?**

*LTFU was observed , one of the reasons we conducted a time to event analysis with total person-time as the denominator to account for LTFU.*

**- The last sentence of the discussion section does not connect well and is kind of confusing. Please revise accordingly.**

*Revised as suggested.*

**Conclusion**

**- This section should summarize your main finding, the implication/s of your findings and recommendation/s. Are the concerns part of your findings? If not, please revise accordingly.**

*The concerns raised owing to increased VLR under UTT are the implications of such a finding.*

**Acknowledgement**

**- Authors can specify why they are grateful to the patients and staff unlike leaving it hanging.**

*For their collaboration.*

**Reviewer #2: Thank you for submitting a manuscript titled,**

**“Changes in access to viral load testing, incidence rates of viral load suppression and rebound following the introduction of the ‘Universal Test and Treat’ guidelines in Cameroon: a retrospective follow-up analysis”. This is a very intriguing subject and adds a lot of knowledge and insights into the UTT approach to HIV elimination. However, I have some concerns which need to be addressed before the article can be published.**

**1. Title**

**I think it would read better if the title was changed to “Trends in viral load access and testing, incidence rates of viral load suppression and rebound following the introduction of the ‘Universal Test and Treat’ guidelines in Cameroon: a retrospective follow-up analysis.” This would inform the analysis approach and enable the researchers to clearly show the yearly changes, rather than just categorizing as Pre-UTT and UTT.**

*We were particularly interested in these two time periods after the introduction of the UTT strategy in Cameroon*.

**2. Methodology.**

**You mentioned that the clinic where the study was conducted started in 2005, but your review included those who initiated ART from 2002, how accurate was this information? And how many had initiated ART between 2002-2005? It would have been better to exclude those who were started on ART before the clinic was opened or include as part of the limitation for the study.**

*We thank you very much indeed for this insight into the manuscript. These are patients who were transferred into the clinic from another clinic.*

**3. Results analysis.**

**It’s better to present a trends analysis from 2002 or 2005 to show how viral coverage and suppression has been changing over time. This will help to explain the micro changes which could have taken place before and after UTT, e.g. changes in ART initiation guidelines and viral load accessibility policies.**

*Given that guidelines have been revised several times across this period, we were focusing the guideline introduced in 2016. We examined the changes in access viral load testing, viral load suppression and viral load rebound.*

**For Viral load suppression, table2, whereas statistically it is correct to say that “the hazard of viral load suppression was 6 times higher in UTT than in pre-UTT”, programmatically it is rather confusing. I therefore suggest that a reanalysis is done with the outcome of interest as viral load non-suppression. This will make it clearer to a programmer.**

*Such a statistical jargon may be useful in the Results section but has been simplified in the Discussion section for the lay person as well. We have however, replaced ‘hazard’ with ‘rate’. A reanalysis to consider VL non-suppression (also known as virological failure or high viral load) will simply inverse the figures. Yet, we have considered VL load non-suppression after a previous suppression in the sub-sectioned on viral load rebound.*
